# Effect of beta-blockers on exacerbation rate and lung function in chronic obstructive pulmonary disease (COPD)

**DOI:** 10.1186/s12931-017-0609-7

**Published:** 2017-06-19

**Authors:** Sean Duffy, Robert Marron, Helen Voelker, Richard Albert, John Connett, William Bailey, Richard Casaburi, J. Allen Cooper, Jeffrey L. Curtis, Mark Dransfield, MeiLan K. Han, Barry Make, Nathaniel Marchetti, Fernando Martinez, Stephen Lazarus, Dennis Niewoehner, Paul D. Scanlon, Frank Sciurba, Steven Scharf, Robert M. Reed, George Washko, Prescott Woodruff, Charlene McEvoy, Shawn Aaron, Don Sin, Gerard J. Criner, the NIH COPD Clinical Research Network and the Canadian Institutes of Health Research

**Affiliations:** 1 Department of Thoracic Medicine and Surgery, Lewis Katz School of Medicine at Temple University, Philadelphia, PA USA; 2 University of Minnesota, Minneapolis, MN USA; 3 Denver Health Medical Center, Denver, CO USA; 4 University of Alabama at Birmingham, Birmingham, AL USA; 5 Los Angeles Biomedical Research Institute at Harbor-UCLA Medical Center, Torrance, CA USA; 6 University of Michigan Health System, Ann Arbor, MI USA; 7 National Jewish Health, Denver, CO USA; 8 Weill Cornell Medical College of Cornell University, New York, NY USA; 9 Univ of California San Francisco, San Francisco, CA USA; 10 Minneapolis VA Medical Center, Minneapolis, MN USA; 11 Mayo Clinic, Rochester, MN USA; 12 University of Pittsburgh Medical Center, Pittsburgh, PA USA; 13 University of Maryland, Baltimore, MD USA; 14 Brigham & Women’s Hospital, Boston, MA USA; 15 HealthPartners, Saint Paul, MN USA; 16 The Ottawa Hospital Research Institute, Ottawa, ON Canada; 17 Providence Heart + Lung Institute, Vancouver, BC Canada; 18 Department of Thoracic Medicine and Surgery, Temple University School of Medicine, 712 Parkinson Pavilion, 3401 North Broad Street, Philadelphia, PA 19140 USA

**Keywords:** COPD, Exacerbation, Beta-blocker

## Abstract

**Background:**

Beta-blockers are commonly prescribed for patients with cardiovascular disease. Providers have been wary of treating chronic obstructive pulmonary disease (COPD) patients with beta-blockers due to concern for bronchospasm, but retrospective studies have shown that cardio-selective beta-blockers are safe in COPD and possibly beneficial. However, these benefits may reflect symptom improvements due to the cardiac effects of the medication. The purpose of this study is to evaluate associations between beta-blocker use and both exacerbation rates and longitudinal measures of lung function in two well-characterized COPD cohorts.

**Methods:**

We retrospectively analyzed 1219 participants with over 180 days of follow up from the STATCOPE trial, which excluded most cardiac comorbidities, and from the placebo arm of the MACRO trial. Primary endpoints were exacerbation rates per person-year and change in spirometry over time in association with beta blocker use.

**Results:**

Overall 13.9% (170/1219) of participants reported taking beta-blockers at enrollment. We found no statistically significant differences in exacerbation rates with respect to beta-blocker use regardless of the prevalence of cardiac comorbidities. In the MACRO cohort, patients taking beta-blockers had an exacerbation rate of 1.72/person-year versus a rate of 1.71/person-year in patients not taking beta-blockers. In the STATCOPE cohort, patients taking beta-blockers had an exacerbation rate of 1.14/person-year. Patients without beta-blockers had an exacerbation rate of 1.34/person-year. We found no detrimental effect of beta blockers with respect to change in lung function over time.

**Conclusion:**

We found no evidence that beta-blocker use was unsafe or associated with worse pulmonary outcomes in study participants with moderate to severe COPD.

## Background

Chronic Obstructive Pulmonary Disease (COPD) is a progressive and debilitating disease that burdens the healthcare system with frequent office visits and hospitalizations. In recent years, studies have examined both traditional treatments for COPD (long acting beta agonists, long acting muscarinic antagonists, and inhaled corticosteroids) [1, 2] as well as drugs usually reserved for cardiovascular disease or infection (statins and azithromycin) [3, 4] with respect to their efficacy in reducing acute exacerbations of COPD (AECOPD).

Beta-blockers are regularly prescribed in patients with cardiovascular disease, a common comorbidity in patients with COPD. Providers have historically been reluctant to treat COPD patients with beta-blockers due to a concern for precipitating bronchospasm. These concerns have been expressed in review articles and practice guidelines that cited case studies of acute bronchospasm in patients treated with non-selective beta blockers [5, 6]. Cardioselective beta-blockers (or beta-1-blockers) have a 20 fold greater affinity for β-1 receptors and less theoretical risk for bronchoconstriction. Within the last decade, studies have highlighted concern for the use of beta-blockers in patients with COPD [6, 7]; however, Cochrane Reviews in 2005 and 2010 concluded that cardioselective beta blocker use in patients with COPD had no significant adverse effects on FEV_1_, respiratory symptoms, or responsiveness to beta-agonist inhaled therapy. Sub-group analysis extended this to patients with severe obstruction as well as those with bronchodilator reversibility demonstrated on spirometry [8].

Multiple retrospective studies have suggested that beta-blockers may reduce the mortality of patients with COPD as well as the risk of AECOPD [9, 10]. Mortality as an endpoint in studies linking COPD and beta-blockers is confounded by difficulty determining whether the benefit of the drug is related to its effects on the lung or on coexistent cardiovascular disease [11, 12]. Examining the relationship between beta-blocker use, serial spirometry and rates of AECOPD may provide a more useful depiction of the effect of these medications on lung disease.

The COPD Clinical Research Network has conducted several randomized, placebo-controlled prospective trials in study participants with COPD assessing rates of AECOPD.

The STATCOPE study showed no benefit attributable to daily simvastatin, whereas the

MACRO trial demonstrated reduced rates of AECOPD with azithromycin treatment [3, 4]. The STATCOPE cohort and the placebo arm of MACRO provide a unique opportunity to analyze the effect of beta-blockers on AECOPD in a group of COPD patients with a fairly high prevalence of cardiovascular comorbidities (MACRO) as compared with a group in which cardiovascular comorbidities were mostly excluded (STATCOPE). Given that beta-blockers are likely to have a greater impact on patients with cardiovascular disease, we hypothesized that beta-blockers would associate with lower rates of COPD exacerbation in the MACRO cohort when compared to the STATCOPE cohort due to a higher burden of underlying cardiovascular comorbidity. That is to say, a population with a higher prevalence of cardiac comorbidities may have a greater symptomatic benefit from treatment with beta-blockers when compared with a population with little or no cardiac comorbid disease.

## Methods

### Patient population

We performed a retrospective review of 1219 study participants who had at least 180 days of follow up from the STATCOPE trial or the placebo arm of the MACRO trial. Entry criteria for the MACRO trial included having a ratio of forced expiratory volume in one second to forced vital capacity (FEV_1_/FVC) < 70% and being at high risk for experiencing an AECOPD as a result of using supplemental oxygen, or being treated with oral glucocorticoids, antibiotics or being hospitalized in the previous year for an AECOPD. Patients also had no exacerbation within 4 weeks of enrollment ^4^. Patients in the azithromycin treatment arm of the MACRO study were excluded due to the significant treatment effect of azithromycin on reducing exacerbation rate. The STATCOPE trial had similar inclusion criteria, but excluded patients who were taking a statin, had contraindication to the use of statins or who were found to have an indication for statin therapy [3]. In both the MACRO and STATCOPE studies exacerbations were defined as “a complex of respiratory symptoms (increased or new onset) of more than one of the following: cough, sputum, wheezing, dyspnea, or chest tightness with a duration of at least 3 days requiring treatment with antibiotics or systemic steroids.”

### Spirometry

Spirometry was obtained at enrollment and at completion of the study. Each cohort’s spirometric data were analyzed for significant changes over time. The MACRO cohort had spirometry performed at enrollment then at either 6 or 12 months. The STATCOPE cohort had spirometry at enrollment then at 12 or 24 months.

### Statistics

The primary study endpoint was rate of acute exacerbation of COPD in each of the four study groups; MACRO on beta-blocker, MACRO off beta-blocker, STATCOPE on beta-blocker and STATCOPE off beta-blocker. COPD exacerbation rates were compared with the use of an event rate ratio; i.e., the number of exacerbations per patient year. Additionally, changes in spirometric data were analyzed over time for each of the four study groups. *P*-values for mean rate of decline were computed by *t*-test. Exacerbation rates were compared among the groups using SAS data analysis software.

## Results

Of the 1219 participants included in our study, 170 (13.9%) reported taking beta-blockers at enrollment. In the STATCOPE cohort 63 of 688 (9.2%) participants were taking beta-blockers along with 107 of 531 (20.2%) in the MACRO placebo arm. The majority of participants in the study had severe or very severe airflow obstruction classified as GOLD stage III or IV. Those taking beta-blockers tended to have a slightly higher FEV_1_, however we found no statistically significant difference between the groups with respect to demographic characteristics, smoking history or spirometric data (Table 1). As expected, the patients in STATCOPE had a significantly decreased rate of cardiac comorbidities as compared with the MACRO cohort (Table 2). Given the disparity in cardiac comorbidities, the vast majority of patients on beta-blockers in the STATCOPE cohort appeared to be taking the medication for hypertension rather than coronary artery disease (CAD) or a history of myocardial infarction (MI); whereas, a significant percentage in the MACRO cohort was taking the medication for CAD or MI (Table 2).

**Table 1:** Demographics, smoking history and spirometry. No statistically significant difference between study groups in within each trial

	Macro -BB	Macro + BB	Statcope -BB	Statcope + BB
N	469	110	625	63
Age - years (SD)	64.6 (8.6)	67.3 (8.0)	62 (8.51)	63.4 (7.9)
Men - N (%)	273 (58.2%)	75 (67.6%)	349 (55.8%)	35 (55.6%)
Race				
% Black	16.8	11.7	20.3	25.4
% White	79.5	82.9	77.1	73
Smoking History				
Pack years - mean (SD)	59.7 (33.7)	59.4 (28.5)	50.0 (26.3)*	49.1 (28.9)
Current Smoker - %	22.4	21.6	30.6*	34.9
Spirometry - %				
GOLD 2	21.8	44.1	34.3	38.1
GOLD 3	42.4	36	33.9	34.9
GOLD 4	35.8	19.8	31.8	27
FEV_1_ - Liters (SD)	1.09 (0.51)	1.31 (0.52)	1.20 (0.57)*	1.30 (0.62)
FEV_1_ - mean % predicted value	38.3	46.8	41.8*	45.8
FVC - Liters (SD)	2.61 (0.89)	2.81 (0.79)	2.68 (0.92)	2.65 (0.95)
FVC - % predicted value	69.4	74.6	70.7	70.3
FEV_1_/FVC	41.8	46.8	44.6	48.8
Chronic Bronchitis - %	44.8	50.5	48.9	52.5

* - *p* < 0.05 when compared with corresponding subgroup MACRO cohort

**Table 2:** Percent of patients self-reporting comorbid conditions by study group

	Macro -BB	Macro + BB	Statcope -BB	Statcope + BB
N	424	107	625	63
Hypertension	193 (46%)	88 (82%)	180 (29%)*	53 (84%)
Diabetes	49 (12%)	25 (23%)	21 (3%)*	3 (5%)*
CAD	54 (13%)	53 (50%)	12 (2%)*	2 (3%)*
MI	40 (9%)	38 (36%)	5 (1%)*	3 (5%)*

* - *p* < 0.05 when compared with corresponding subgroup MACRO cohort

Patients in the STATCOPE cohort taking beta-blockers had the lowest rate of AECOPD (1.14/person-year, 95% CI = 0.81–1.46), followed by patients in STATCOPE not taking beta-blockers (1.34/person-year, 95% CI = 1.22–1.46). In the MACRO cohort the rate of AECOPD was higher when compared with STATCOPE, but nearly identical with respect to beta-blocker usage within the cohort (Table 3). We found no statistically significant difference between patients taking beta-blockers and the corresponding cohort off beta-blockers.

**Table 3:** AECOPD per person year by study group

Statcope	N	Exacerbation rate per person-year (95% CI)
+BB	63	1.14 (0.81,1.46)
−BB	625	1.34 (1.22,1.46)
Macro		
+BB	107	1.72 (1.37,2.08)
−BB	424	1.71 (1.53,1.88)

**Fig. 1:**
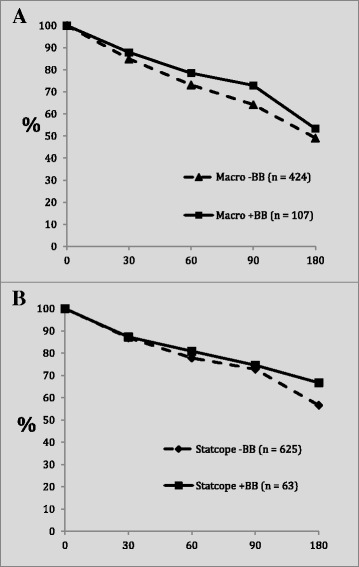
Panel (**a**) - Percent free of exacerbation by beta-blocker use in the Macro placebo arm at 0, 30, 60, 90 and 180 days. Panel (**b**) – Percent free of exacerbation by beta-blocker use in the STATCOPE trial

Percent of subjects free of exacerbation over time is depicted in Fig. 1. In both the MACRO and STATCOPE cohorts, the patients taking beta-blockers had a higher percentage of patients free of exacerbation though this difference did not reach statistical significance in either group. Of note, there was no patient dropout during this period as all patients had at least 180 days of follow up.

Additionally, we studied the change in spirometric data over time for all patients (1063/1219, 87.2%) who had follow-up spirometry. The data shows that the presence of beta-blocker medications had no clinically or statistically significant effect on the change in airflow limitation in this cohort.

## Discussion

We found no harmful effect of beta-blockers with respect to change in FEV_1_ over time and no statistically significant difference in the rate of acute exacerbation of COPD in an at-risk population, regardless of the presence of cardiac comorbidity.

Other retrospective studies have shown beta-blockers to be beneficial in patients with COPD but those observations may represent cardiovascular benefits of beta-blockers rather than pulmonary specific improvements in COPD symptoms or severity.

One recent Swedish nationwide observational study concluded that patients with COPD discharged on a beta-blocker after an MI had a lower all-cause mortality compared with those not discharged on a beta-blocker [13]. However, a large retrospective study showed that patients with severe COPD or asthma had no mortality benefit from taking beta-blockers after MI [14]. Mortality has been shown to be improved in COPD patients specifically taking beta-blockers as monotherapy for hypertension [15]. The reduced prevalence of cardiac comorbidity in the STATCOPE cohort provided an opportunity to compare the effectiveness of beta-blockers in a COPD population with (MACRO) and without (STATCOPE) self-reported cardiac disease. We found no significant difference in rates of AECOPD with respect to beta-blocker use in the cohort with increased cardiac comorbidities. However, there was a slightly lower rate of AECOPD in the patients taking beta-blockers in the STATCOPE cohort and the percent free of exacerbation at 90 and 180 days was higher in patients taking beta-blockers in each cohort. Our inability to detect a statistically significant difference between patients with versus without cardiac disease may be a result of the relatively low number of patients who were taking beta-blockers and reported cardiac comorbidities. Importantly, we did find that COPD patients taking beta-blocker medications did no worse with respect to exacerbation or change in spirometry over a relatively long follow up period when compared with patients who were not taking beta-blockers (Table 4). In accordance with prior studies [13–16], this finding provides further proof that beta-blockers are safe to use in COPD patients.

**Table 4:** Change in spirometry over time with respect to beta-blocker use

Statcope	∆FEV1 in mL (SD)	∆ %FEV1 (SD)	∆ FVC in mL (SD)	∆ %FVC (SD)
+BB (N = 54)	−13.1 (268.8)	0.33 (9.7)	−21.7 (382.9)	0.59 (10.2)
−BB (N = 531)	−53.9 (241.3)	−1.24 (8.96)	−88.8 (444.4)	−1.43 (12)
*P*- value	0.29	0.25	0.23	0.18
Macro				
+ BB (N = 96)	−13.9 (233.9)	−0.55 (8.61)	n/a	−0.52 (12.3)
−BB (N = 381)	−12.7 (196)	−0.31 (6.5)	n/a	−0.47 (12.4)
*P*- value	0.96	0.76		0.97

*n/a* not included as data reported for only 4 participants

Proposed mechanisms by which beta-blockers may have an effect on the COPD process and potentially decrease exacerbation frequency include reduction of ischemic burden and tempering the sympathetic nervous system. COPD has been associated with systemic inflammation, and it has been proposed that the negative effects of neurohumoral activation (such as inflammation, cachexia) can contribute to the cycle of COPD exacerbation and pathophysiology. Beta blockade theoretically could have an impact on neuro-humoral activation and COPD. [17] In addition, animal models have shown that beta-blockers given chronically can increase the density of beta-receptors and reduce airway responsiveness in mice with asthma [18]. Those on beta-blockers may have had more effective blood pressure control and a reduction in complications of less optimally treated diastolic dysfunction which has been linked to the development of acute exacerbations [19].

Other observational studies have shown conflicting results. For instance, Bhatt and colleagues evaluated a cohort of over 3000 patients from the COPDGene cohort and found that patients taking beta-blockers had a decreased incidence on AECOPD. This association held true for severe exacerbations as well as mild to moderate exacerbations [20]. Short et.al. performed a retrospective review of COPD patients on and off beta-blockers and showed that patients taking beta-blockers had lower rates of AECOPD and mortality regardless of the inhaled pulmonary medication regimen in each group [21]. However, in both of these trials, patients had less severe airflow obstruction in comparison to our population (Table 5). Our inability to find a significant effect of beta-blockers may be secondary to the severity of COPD in this cohort. These patients were enrolled due to an increased risk for COPD exacerbation and had relatively severe obstruction on spirometry. This high burden of respiratory disease may overshadow the relatively lesser burden of cardiovascular disease in this population. We also found a high prevalence of chronic bronchitis in each cohort, which was not reported in the other studies, but may have influenced the rate of exacerbation and limited the efficacy of beta-blocker therapy.

**Table 5:** Demographic comparison of retrospective studies on beta-blocker use in COPD

Study	Bhatt et al. [20]	Rutten et al. [11]	Short et al. [21]	Van Gestel et al. [16]	Current Study	
					MACRO^4^	STATCOPE^3^
N (on Beta blockers)	474	665	796	462	110	63
Age	66.8	64.7	69.8	69	67.3	63.4
% Men	60.1%	49.8%	42.7%	82.0%	67.6%	55.6%
Mean Pack years	56.8	n/a	44.3	n/a	59.4	49.1
% Current Smoker	n/a	34.6%	n/a	35.0%	21.6%	34.9%
FEV1 (L)	1.5	n/a	n/a	n/a	1.31	1.3
FEV1 mean % predicted	53.2%	n/a	65%	n/a	46.8%	45.8%
% GOLD 3/4	38.4%	n/a	n/a	n/a	55.8%	61.9%
%Chronic bronchitis	n/a	n/a	n/a	n/a	50.5%	52.5%
% CAD or revasc	44.7%	38.3% (IHD)	70% (CV dz)	25%	50%	3%
% Diabetes	24.1%	24.1%	21%	17%	23%	5%
% Hypertension	84.4%	66.8%	n/a	49%	82%	84%
% History of MI	n/a	9.6%	n/a	33%	38%	5%

*n/a* data not available

Additionally, the expected outcome of beta-blockers having a greater effect on the cohort with cardiac comorbidity (MACRO) may not have been present due to baseline differences in the two groups. The MACRO cohort on beta-blockers trended to be an older population, with an increased number of pack-years which may be reflective of a sicker patient population when compared with the STATCOPE cohort on beta-blockers (Table 1).

## Study limitations

The relatively low number of patients taking beta-blockers at enrollment in these studies limited our ability to show a statistically significant difference between the groups with respect to demographic and baseline spirometry (Table 1). Medications and comorbidities were self-reported by the patients at enrollment. Information regarding dosage and specific type of beta-blocker (i.e. beta-1 selective or nonselective) medication was not available. Though there is inherent weakness in the design of all retrospective analyses, the data presented are derived from well-constructed, prospective, large multicenter trials. Despite the limitations, our study is the first to compare the efficacy of beta-blockers in a COPD population with higher versus lower prevalence of cardiac comorbidities.

## Conclusion

We found no evidence that beta-blockers significantly affected the rate of AECOPD regardless of whether the patients did or did not report cardiac comorbidities. We did find that beta-blockers had no detrimental effect on lung function in a population that was at increased risk for AECOPD. Because previous observational studies have reported conflicting data, the question will require a large, prospective and randomized trial to determine the benefits of beta-blocker therapy in COPD patients.
